# Upper Bound for the Ground State Energy of a Dilute Bose Gas of Hard Spheres

**DOI:** 10.1007/s00205-024-02049-w

**Published:** 2024-10-21

**Authors:** Giulia Basti, Serena Cenatiempo, Alessandro Giuliani, Alessandro Olgiati, Giulio Pasqualetti, Benjamin Schlein

**Affiliations:** 1 Dipartimento di Matematica, Sapienza Università di Roma, Piazzale Aldo Moro 5, 00185 Rome, Italy; 2 Gran Sasso Science Institute, Viale Francesco Crispi 7, 67100 L’Aquila, Italy; 3 Università degli Studi Roma Tre, L.go S. Leonardo Murialdo 1, 00146 Rome, Italy; 4 Institute of Mathematics, University of Zurich, Winterthurerstrasse 190, 8057 Zurich, Switzerland; 5 Present Address: Istituto di Matematica, Politecnico di Milano, Piazza Leonardo da Vinci 32, 20133 Milan, Italy

## Abstract

We consider a gas of bosons interacting through a hard-sphere potential with radius $\mathfrak {a}$ in the thermodynamic limit. We derive an upper bound for the ground state energy per particle at low density. Our bound captures the leading term $4\pi \rho \mathfrak {a}$ and shows that corrections are smaller than $C \rho \mathfrak {a} (\rho {{\mathfrak {a}}}^3)^{1/2}$, for a sufficiently large constant $C > 0$. In combination with a known lower bound, our result implies that the first sub-leading term to the ground state energy of a dilute gas of hard spheres is, in fact, of the order $\rho \mathfrak {a}(\rho {{\mathfrak {a}}}^3)^{1/2}$, in agreement with the Lee–Huang–Yang prediction.

## Introduction and Main Result

In recent years, there has been substantial progress in the mathematical understanding of the low-energy properties of dilute Bose gases. In the Gross–Pitaevskii regime, in which *N* particles on the unit torus interact through a repulsive potential with range and scattering length of the order 1/*N*, the ground state energy and the low-energy excitation spectrum have been determined in [6], up to errors vanishing as $N \rightarrow \infty$, under the assumption that the interaction potential $V \in L^3 (\mathbb {R}^3)$ is repulsive, radial and of compact support. The proof applies optimal estimates on the number and the energy of excitations of the Bose–Einstein condensate that have been previously established in [4, 5]. Recently, a new derivation of these precise bounds has been proposed in [21]. The results of [6] have been extended to systems of bosons trapped by an external potential (again in the Gross–Pitaevskii regime) in [7, 8, 33, 35]. They have been also generalized to the two-dimensional setting in [10, 11]. An upper bound on the ground state energy has been shown in [1] for particles in the Gross–Pitaevskii regime, interacting through a hard-sphere potential. These results extend leading order estimates on the ground state energy that have been known since [30, 31] and previous proofs of Bose–Einstein condensation obtained in [27, 28, 34].

In the thermodynamic limit, where *N* particles interacting through a repulsive potential *V* with scattering length $\mathfrak {a}$ are confined on a torus $\Lambda$ and $N, |\Lambda | \rightarrow \infty$ with fixed density $\rho = N / |\Lambda |$, the ground state energy per particle has been predicted by Lee–Huang–Yang in [25] to satisfy

1.1 $$\begin{aligned} \lim _{\begin{array}{c} N, |\Lambda | \rightarrow \infty : \\ \rho = N/|\Lambda | \end{array}} \frac{E_N}{N} = 4 \pi \mathfrak {a} \rho \Big [ 1 + \frac{128}{15 \sqrt{\pi }} (\rho \mathfrak {a}^3)^{1/2} + ... \Big ] \end{aligned}$$

in the dilute regime $\rho \mathfrak {a}^3 \rightarrow 0$. The validity of the leading order term on the r.h.s. of (1.1) was proven in [14] (upper bound) and [32] (lower bound). An upper bound matching (1.1) was later shown in [38] for sufficiently regular interaction potentials (improving an estimate previously shown in [15]). Recently, a simpler proof applying to every repulsive and radial $V \in L^3 (\mathbb {R}^3)$ was obtained in [2]. A lower bound to the ground state energy matching (1.1) was established in [18] for integrable potential and then in [19], also for hard-sphere interactions. In [9, 12], the Lee–Huang–Yang formula (1.1) is proven, following a strategy proposed in [26], under the assumption that the reduced densities associated with the ground state wave function satisfy certain relations. Although these relations have not yet been rigorously verified, they appear to capture the behaviour of Bose gases also beyond the dilute regime. Recently, a second order expansion for the ground state energy per particle of two dimensional Bose gases has been proven in [17] for all positive potentials with finite scattering length. The asymptotics of the ground state energy of dilute Fermi gases was first studied in [29]; for recent progress see [16, 20, 23, 24].

Despite these advances, the derivation of an upper bound for the ground state energy resolving the Lee–Huang–Yang corrections in (1.1) for hard-sphere potentials remains open. In the present work, we make a step in this direction, providing a simple proof of the fact that the ground state energy per particle for hard-spheres in the thermodynamic limit is given by the leading term on the r.h.s. of (1.1), up to errors that are bounded above by $C \rho {\mathfrak a }(\rho {{\mathfrak {a}}}^3)^{1/2}$, in the limit $\rho {\mathfrak a}^3 \rightarrow 0$. Our result improves an upper bound obtained by Dyson [14], where the error was of the order $\rho \mathfrak {a}(\rho {{\mathfrak {a}}}^3)^{1/3}$.

We consider *N* hard spheres moving in the box $\Lambda = [ - L/2 ; L/2 ]^3$, with periodic boundary conditions. We are interested in the limit $N,L \rightarrow \infty$ at fixed density $\rho = N / |\Lambda |$. We define the ground state energy by where the infimum is taken over all $\Psi _N \in L^2_s (\Lambda ^N)$, the subspace of $L^2 (\Lambda ^N)$ consisting of functions that are symmetric w.r.t. permutations of the *N* particles, satisfying the hard-sphere condition

1.2 $$\begin{aligned} \Psi _N (x_1, \ldots , x_N) = 0 \qquad \text{ if } \text{ there } \text{ exist } i, j \in \{ 1, \ldots , N \},\, i \ne j\quad \text{ with } |x_i - x_j| \le \mathfrak {a}\,. \nonumber \\ \end{aligned}$$

Here $|x_i - x_j|$ denotes the distance on the torus between $x_i$ and $x_j$.

### Theorem 1.1

There exists $C > 0$ such that

$$ \lim _{\begin{array}{c} N,L \rightarrow \infty : \\ N/ |\Lambda | = \rho \end{array}} \frac{E^\text {hs}_N}{N} \le 4\pi \rho \mathfrak {a} \Big [ 1 + C (\rho \mathfrak {a}^3)^{1/2} \Big ] $$

for all $\rho \mathfrak {a}^3 > 0$ small enough.

## The Trial State

In order to show Theorem 1.1, we consider a wave function having the form

2.1 $$\begin{aligned} \Psi _N (x_1, \ldots , x_N) = \prod _{i<j}^N f_\ell (x_i - x_j)\,. \end{aligned}$$

Such trial states have been first used in [3, 13, 22]; for this reason we will refer to the product on the r.h.s. of (2.1) as a Bijl–Dingle–Jastrow factor. In (2.1), $f_\ell$ is chosen to describe correlations between particles, up to a distance $\ell \ll L$. More precisely, we choose $f_\ell$ to be the ground state solution of the Neumann problem

$$\begin{aligned} \left\{ \begin{aligned} -\Delta f_\ell&= \lambda _\ell f_\ell \\ f_\ell (x)&= 0 \qquad \text {for all } |x| < \mathfrak {a} \\ \partial _r f_\ell (x)&= 0 \qquad \text {if } |x| = \ell \end{aligned} \right. \end{aligned}$$

on the ball $B_\ell = \{ x \in \mathbb {R}^3 : |x| \le \ell \}$, associated with the smallest eigenvalue $\lambda _\ell$. We normalize $f_\ell$ by requiring that $f_\ell (x) = 1$ for $|x| = \ell$. We extend $f_\ell$ to $\Lambda$, setting $f_\ell (x) =1$ for all $|x| \ge \ell$. We then have

2.2 $$\begin{aligned} -\Delta f_\ell (x) = \lambda _\ell \chi _\ell (x) f_\ell (x) \end{aligned}$$

where $\chi _\ell$ denotes the characteristic function of the ball $B_\ell$. The proof of the following lemma can be found in [1, Lemma 2.1] (it is easy to translate the bounds on $\omega _\ell = 1 - f_\ell$ stated in [1] into the estimates for $u_\ell = 1- f^2_\ell$ appearing here):

### Lemma 2.1

For $\mathfrak {a} \ll \ell$, we have

$$\begin{aligned} \lambda _\ell =\frac{3 \mathfrak {a}}{\ell ^3} \Big [ 1+\mathcal {O} (\mathfrak {a} / \ell ) \Big ]\,.\end{aligned}$$

Moreover, $0 \le f_\ell (x) \le 1$ for all $x \in \Lambda$ and, defining $u_\ell (x) = 1 - f^2_\ell (x)$, we find

$$\begin{aligned} 0 \le u_\ell (x) \le C \mathfrak {a} \frac{\chi _\ell (x)}{|x|} , \qquad |\nabla u_\ell (x) | \le C \mathfrak {a} \frac{\chi _\ell (x)}{|x|^2}\,. \end{aligned}$$

Since (2.1) satisfies the hard-core condition (1.2), we immediately obtain that

$$\begin{aligned} E_N^\text {hs} \le \sum _{j=1}^N \frac{\langle \Psi _N, -\Delta _{x_j} \Psi _N \rangle }{\Vert \Psi _N \Vert ^2} \,. \end{aligned}$$

For $j=1, \ldots , N$, we compute

$$ \begin{aligned} -\Delta _{x_j} \Psi _N (x_1, \ldots , x_N)&= \; \sum _{i \not = j}^N \frac{-\Delta f_\ell (x_j - x_i)}{f_\ell (x_j - x_i)} \Psi _N (x_1, \dots , x_N) \\&\quad - \sum ^N_{\begin{array}{c} i,m \not = j \\ i \not = m \end{array}} \frac{\nabla f_\ell (x_j - x_i)}{f_\ell (x_j -x_i)} \cdot \frac{\nabla f_\ell (x_j - x_m)}{f_\ell (x_j - x_m)} \Psi _N (x_1, \dots , x_N)\,. \end{aligned} $$

From (2.2), we obtain

$$ \begin{aligned} \langle \Psi _N, -\Delta _{x_j} \Psi _N \rangle =\;&\sum _{i \not = j}^N \int \lambda _\ell \chi _\ell (x_j- x_i) |\Psi _N (x_1 \dots , x_N)|^2 \, \text {d}x_1 \dots \text {d}x_N \\  &- \sum _{\begin{array}{c} i,m \not = j \\ i \not = m \end{array}} \int \frac{\nabla f_\ell (x_j - x_i)}{f_\ell (x_j -x_i)} \cdot \frac{\nabla f_\ell (x_j - x_m)}{f_\ell (x_j - x_m)}\\  &\hspace{56.9055pt}\times |\Psi _N (x_1, \ldots , x_N)|^2\, \text {d}x_1 \dots \text {d}x_N \,. \end{aligned} $$

For $i,j \in \{ 1, \ldots , N \}$, we write $V_{ij} = 2 \lambda _\ell \chi _\ell (x_i - x_j)$ and $f_{ij} = f_\ell (x_i - x_j)$. With this short-hand notation (and omitting the measure $\textrm{d}x_1 \dots \textrm{d}x_N$ from all integrals), we find

$$\begin{aligned} \frac{E_N^\text {hs}}{N} \le \frac{(N-1)}{2} \frac{\int V_{12} \prod _{i<j}^N f_{ij}^2}{\int \prod _{i<j}^N f_{ij}^2} - \frac{(N-1)(N-2)}{6} \frac{\int \frac{\nabla f_{13}}{f_{13}} \cdot \frac{\nabla f_{23}}{f_{23}} \prod _{i<j}^N f_{ij}^2}{\int \prod _{i<j}^N f_{ij}^2}\,. \end{aligned}$$

The two terms on the r.h.s. of the last equation will be considered in the next two propositions, whose proof is deferred to the next sections.

### Proposition 2.2

Fix $\ell = c \, (\rho {{\mathfrak {a}}})^{-1/2}$ for a sufficiently small constant $c > 0$. Then there is a constant $C > 0$ such that

2.3 $$\begin{aligned} \limsup _{\begin{array}{c} N,|\Lambda | \rightarrow \infty : \\ N/|\Lambda | = \rho \end{array}} \frac{N}{2} \frac{\int V_{12} \prod _{i<j}^N f_{ij}^2}{\int \prod _{i<j}^N f_{ij}^2} \le 4 \pi {{\mathfrak {a}}}\rho + C \rho {{\mathfrak {a}}}(\rho {{\mathfrak {a}}}^3)^{1/2} \end{aligned}$$

for all $\rho \mathfrak {a}^3 > 0$ small enough.

### Proposition 2.3

Fix $\ell = c \, (\rho {{\mathfrak {a}}})^{-1/2}$ for a sufficiently small constant $c > 0$. Then there is a constant $C > 0$ such that

2.4 $$\begin{aligned} \limsup _{\begin{array}{c} N,|\Lambda | \rightarrow \infty : \\ N / |\Lambda | = \rho \end{array}} \left| N^2 \frac{\int \frac{\nabla f_{13}}{f_{13}} \cdot \frac{\nabla f_{23}}{f_{23}} \prod _{i<j}^N f_{ij}^2}{\int \prod _{i<j}^N f_{ij}^2}\right| \le C \rho {{\mathfrak {a}}}(\rho {{\mathfrak {a}}}^3)^{1/2}\ \end{aligned}$$

for all $\rho \mathfrak {a}^3 > 0$ small enough.

From Propositions 2.2 and 2.3 (and from the existence of the thermodynamic limit for the energy per particle $E_N^\text {hs}/N$), we immediately conclude that there exists $C > 0$ such that

$$ \lim _{\begin{array}{c} N,|\Lambda | \rightarrow \infty : \\ N / |\Lambda | = \rho \end{array}} \frac{E_N^\text {hs}}{N} \le 4 \pi {{\mathfrak {a}}}\rho \left[ 1 + C (\rho {{\mathfrak {a}}}^3)^{1/2} \right] $$

for all $\rho \mathfrak {a}^3 > 0$ small enough. This completes the proof of Theorem 1.1.

The proof of Propositions 2.2 and 2.3 is based on rewriting $f^2_{ij}=1-u_{i,j}$ in the Bijl–Dingle–Jastrow factors appearing in the numerator and denominator of (2.3) and (2.4), on expanding it in powers of $u_{i,j}$ and on exploiting precise cancellations between the numerator and the denominator. The various terms in the expansion can be graphically represented as diagrams in which vertices represent particles’ labels and lines connecting vertices correspond to factors $u_{i,j}$, or to the observables $V_{12}$ or $\nabla f_{1j}/f_{1j}$ with $j=2,3$. There are two kinds of cancellations between the diagrams at the numerator and at the denominator. One is standard, and is at the very root of the cluster expansion method: all disconnected diagrams cancel between numerator and denominator, and one is left with an expansion over connected diagrams only. This cancellation is not enough for proving that the error term in (2.3) is of relative order $(\rho {{\mathfrak {a}}}^3)^{1/2}$, but ‘just’ $(\rho {{\mathfrak {a}}}^3)^{1/3}$, the same as the error term in Dyson’s upper bound [14]. In order to go beyond this one needs to identify additional, more subtle, cancellations. Explicit computations at low orders show that all tree diagrams cancel between numerator and denominator: this suggests that only connected diagrams *with loops* should survive (here and in the following we will denote with *loop* a set of lines forming a closed non-self-interacting path). In fact, all ‘reducible’ diagrams (namely diagrams which remain connected after the removal of any edge or vertex) cancel at the first few orders, but the cancellation of trees is sufficient to obtain an error term comparable with the Lee–Huang–Yang correction. The cancellation of reducible diagrams was already noticed by Jastrow, see [22, Eqs. (11)–(11c)] and is explicitly discussed in [36, below Eq. (3.6) and Fig. 8], even though not proved systematically. Its rigorous proof has been obtained much more recently within a convergent cluster expansion scheme in the canonical ensemble [37]. In this paper, instead of using a standard cluster expansion, we find it more convenient to expand the variables one by one both in the numerator and in the denominator, choosing the order of the expansion large enough for the truncation errors to be small. At each step of this partial expansion, we estimate contributions associated with diagrams having at least one loop and we isolate trees that disentangle from the remaining Bijl–Dingle–Jastrow factor. The contribution of these diagrams cancels out when we combine the estimates we obtain for the numerator and the denominator.

The proof of Propositions 2.2 and 2.3 relies heavily on the fact that $c>0$ in the definition $\ell =c(\rho \mathfrak {a})^{-1/2}$ is chosen small enough (see for example (3.18)). This assumption ultimately ensures that our partial expansion of the Bijl–Dingle–Jastrow factor converges. To capture the second order correction on the r.h.s. of (1.1), we would need to include correlations on slightly longer scales, choosing $\ell = (\rho a)^{-1/2 - \varepsilon }$, which unfortunately would lead to divergencies. In the Gross–Pitaevskii regime, this issues could recently be solved in [1], combining a Bijl–Dingle–Jastrow factor on short scales with a quasi-free state on larger scales.

## Proof of Proposition 2.2

We set $\ell = c \, (\rho \mathfrak {a})^{-1/2}$ for a sufficiently small constant $c > 0$ to be specified later on. Then

3.1 $$\begin{aligned} \rho \mathfrak {a} \ell ^2 = c^2 \ll 1 \, . \end{aligned}$$

Let us also define $u_{i,j}=u_\ell (x_i-x_j)=1-f^2_{ij}$ for any $1\le i<j\le N$. We will use, several times, the bounds

3.2 $$\begin{aligned} \prod _{j=r}^N f^2_{ij}\le \;&1 +\sum _{m=1}^M(-1)^m \sum _{r\le j_1<\dots <j_m \le N} u_{i,j_1}\dots u_{i,j_m} \end{aligned}$$

3.3 $$\begin{aligned} \prod _{\begin{array}{c} j=r \end{array}}^N f^2_{ij}\ge \;&1 +\sum _{m=1}^{{M+1}} (-1)^m\sum _{r\le j_1<\dots <j_m \le N} u_{i,j_1}\dots u_{i,j_m} \end{aligned}$$

which are valid for any $1\le i <r \le N$ and each $M\ge 0$ even (since $u_\ell \ge 0$), as well as their immediate consequence

3.4 $$\begin{aligned}  &   \Big | \prod _{j=r}^N f^2_{ij} - 1-\sum _{m=1}^M (-1)^{m} \sum _{r \le j_1< \dots<j_{m} \le N} u_{i,j_1}\dots u_{i,j_{m}} \Big | \nonumber \\  &   \quad \le \sum _{r \le j_1<\dots < j_{M+1} \le N} \, u_{i,j_1}\dots {u_{i,j_{M+1}} } \end{aligned}$$

which is valid for any $1\le i<r \le N$ and $M\ge 0$. The validity of (3.2) and (3.4) follows by induction using $0\le u_\ell \le 1$.

We introduce the notation

3.5 $$\begin{aligned} I_{N-k} = \int \prod _{k+1 \le i < j \le N} f_{ij}^2 \, \textrm{d}x_{k+1} \dots \textrm{d}x_N \end{aligned}$$

for $k =0,1, \ldots , N-2$. We observe that $I_{N} \le I_{N-1} |\Lambda |.$ At the same time, $u (x) = 1- f_\ell ^2 (x)$ using Lemma 2.1 to estimate $\Vert u_\ell \Vert _1 \le C \mathfrak {a} \ell ^2$, we find $I_{N} \ge I_{N-1} (|\Lambda | - C N \Vert u_\ell \Vert _1) \ge |\Lambda | I_{N- 1} (1 - C \rho \mathfrak {a} \ell ^2) \ge |\Lambda | I_{N- 1} / 2$ choosing $c > 0$ in (3.1) small enough. Repeating the same argument, we obtain

3.6 $$\begin{aligned} 2^{-k} |\Lambda |^k I_{N-k} \le I_{N} \le |\Lambda |^k I_{N-k} \end{aligned}$$

for all $k\in \mathbb {N}$ with $k \le N-2$.

We consider the numerator on the l.h.s. of (2.3). We isolate the term $f_{12}^2$ and we expand the remaining $x_1$-dependence in the Bijl–Dingle–Jastrow factor. With (3.2), we obtain

3.7 $$\begin{aligned} \begin{aligned}&\int V_{12} \prod _{1 \le i,j \le N} f_{ij}^2 \\  &\le \int V_{12} f_{12}^2 \Big [ 1 - \sum _{3 \le r_1 \le N} u_{1,r_1} + \dots + \sum _{3 \le r_1< r_2< \dots< r_{M} \le N} u_{1,r_1} \dots u_{1, r_{M}} \Big ] \prod _{2 \le i< j \le N} f_{ij}^2 \\  &= \sum _{m_1 = 0}^{M} (-1)^{m_1} \sum _{3 \le r_1< r_2< \dots< r_{m_1} \le N} \int V_{12} f_{12}^2 u_{1,r_1} \dots u_{1, r_{m_1}} \prod _{2 \le i< j \le N} f_{ij}^2 \\  &= \sum _{m_1 = 0}^{M} (-1)^{m_1} {N-2 \atopwithdelims ()m_1} \int V_{12} f_{12}^2 u_{1,3} u_{1,4} \dots u_{1,m_1+2} \prod _{2 \le i < j \le N} f_{ij}^2\,\,. \end{aligned}\nonumber \\ \end{aligned}$$

Here, and similarly below, we use the convention that, if $m_1 = 0$, there is no factor of $u_\ell$ in the integral. Next, we expand the $x_2$-dependence in the Bijl–Dingle–Jastrow factor. Stopping the expansion at $m_2=M-m_1$ and using (3.2) or (3.3) depending on the parity of $M-m_1$ we find

3.8 $$\begin{aligned} \begin{aligned}&\int V_{12} \prod _{1 \le i,j \le N} f_{ij}^2 \\  &\quad \le \sum _{m_1 = 0}^{M} (-1)^{m_1} {N-2 \atopwithdelims ()m_1} \sum _{m_2 = 0}^{M - m_1} (-1)^{m_2} \\  &\qquad \times \sum _{3 \le r_1< \dots< r_{m_2} \le N} \int V_{12} f_{12}^2 u_{1,3} \dots u_{1,m_1+2} \, u_{2,r_1} \dots u_{2, r_{m_2}} \prod _{3 \le i < j \le N} f_{ij}^2 \,. \end{aligned}\nonumber \\ \end{aligned}$$

Furthermore, we get rid of the contribution of the loops, namely of the terms where there exists at least one index $i \in \{1, \ldots , m_2\}$ with $r_i \in \{3, \ldots , m_1+2$}. We find

3.9 $$\begin{aligned} \begin{aligned}&\int V_{12} \prod _{1 \le i,j \le N} f_{ij}^2 \\  &\quad \le \sum _{m_1 = 0}^{M} (-1)^{m_1} {N-2 \atopwithdelims ()m_1} \sum _{m_2 = 0}^{M - m_1} (-1)^{m_2} {N-2-m_1 \atopwithdelims ()m_2} \\&\qquad \times \int V_{12} f_{12}^2 u_{1,3} \dots u_{1,m_1+2} u_{2,m_1+3} \dots u_{2, m_1 + m_2 + 2} \prod _{3 \le i < j \le N} f_{ij}^2 \\&\qquad + \mathcal {E}_{\text {loops},2} \end{aligned} \end{aligned}$$

where (denoting by *k* the number of loops)

$$ \begin{aligned} \mathcal {E}_{\text {loops},2}&= \sum _{m_1 = 1}^{M} (-1)^{m_1} {N-2 \atopwithdelims ()m_1} \sum _{m_2 = 1}^{M - m_1} (-1)^{m_2} \sum _{k=1}^{\min (m_1, m_2)} {m_1 \atopwithdelims ()k}{N-2 - m_1 \atopwithdelims ()m_2 -k } \\&\quad \times \int V_{12} f^2_{12} u_{1,3} \ldots u_{1,k+2} u_{2,3} \ldots u_{2,k+2}\\&\quad \times u_{1, k+3} \ldots u_{1, m_1+2} u_{2, m_1 +3} \ldots u_{2, m_1+ m_2 +2-k} \prod _{3 \le i <j \le N} f^2_{ij}\,. \end{aligned}$$

From Lemma 2.1, we have $u\ell (x) \le C\mathfrak {a} \chi _\ell (x) / |x|$. Thus, we can estimate

$$ \begin{aligned}&\Big | \int V_{12} f^2_{12} u_{1,3} \ldots u_{1,k+2} u_{2,3} \ldots u_{2,k+2} \, \text {d}x_1\cdots \text {d}x_{k+2} \Big | \\  &\quad \le C^{k} {{\mathfrak {a}}}^{2k} {\lambda _\ell } |\Lambda | \int \chi (|x|\le \ell )\\  &\qquad \times \prod _{j=1}^k \frac{\chi (|y_j|\le \ell )}{|y_j|} \frac{\chi (|x+y_j|\le \ell )}{|x+y_j|} \, \text {d}x dy_1 \dots dy_k \le C {{\mathfrak {a}}}|\Lambda | (C {{\mathfrak {a}}}^2 \ell )^{k} \end{aligned}$$

for a constant $C > 0$ independent of all parameters. Using again the bound in Lemma 2.1 to show that $\Vert u_\ell \Vert _1 \le C \mathfrak {a} \ell ^2$ and (3.6), this implies that

$$ \begin{aligned} N \mathcal {E}_{\text{ loops },2}&\le C \mathfrak {a} |\Lambda | \sum _{m_1 = 1}^M \frac{1}{m_1!} \sum _{m_2 = 1}^{M-m_1} \sum _{k=1}^{\min (m_1, m_2)} \left( {\begin{array}{c}m_1\\ k\end{array}}\right) \frac{1}{(m_2-k)!} \\  &\hspace{1cm} \times N^{m_1 + m_2 +1 -k} \Vert u_\ell \Vert _1^{m_1 + m_2 -2k} (C \mathfrak {a}^2 \ell )^k I_{N-(m_1 + m_2 +2-k)} \\  &\le C \rho \mathfrak {a} I_N \sum _{m_1=1}^M \frac{1}{m_1!} \sum _{m_2= 1}^{M-m_1} \sum _{k=1}^{\min (m_1, m_2)} \left( {\begin{array}{c}m_1\\ k\end{array}}\right) \\  &\quad \times \frac{1}{(m_2-k)!} (C \rho \mathfrak {a} \ell ^2)^{m_1 + m_2 - 2k} (C \rho \mathfrak {a}^2 \ell )^k \ \end{aligned} $$

with an appropriate choice of the constant $C> 0$. Exchanging the sums over *k* and $m_2$, and shifting $m_2 \rightarrow m_2 + k$, we arrive at

$$ \begin{aligned} N \mathcal {E}_{\text{ loops },2}&\le C \rho \mathfrak {a} I_N \sum _{m_1=1}^M \sum _{k=1}^{m_1} \sum _{m_2 = 0}^{M-m_1-k} \left( {\begin{array}{c}m_1\\ k\end{array}}\right) \frac{1}{m_2!} (C \rho \mathfrak {a} \ell ^2)^{m_1 + m_2 - k} (C \rho \mathfrak {a}^2 \ell )^k \\  &\le C \rho \mathfrak {a} I_N \sum _{m_1=1}^M \sum _{k=1}^{m_1} \left( {\begin{array}{c}m_1\\ k\end{array}}\right) (C \rho \mathfrak {a} \ell ^2)^{m_1 - k} (C \rho \mathfrak {a}^2 \ell )^k \\  &\le C \rho \mathfrak {a} I_N \sum _{m_1=1}^M \sum _{k=1}^{m_1} \left( {\begin{array}{c}m_1\\ k\end{array}}\right) (C \rho \mathfrak {a} \ell ^2)^{m_1} (C \mathfrak {a} / \ell )^k \le C \rho \mathfrak {a} (\rho \mathfrak {a}^2 \ell ) I_N\,. \end{aligned} $$

In (3.9), we also separate terms with $m_1 + m_2 = 0$ (in this case, there is only the term with $m_1 = m_2 = 0$), where the Bijl–Dingle–Jastrow factor is no longer entangled with the observable, from the other terms. We obtain

3.10 $$\begin{aligned} \begin{aligned} \int V_{12}&\prod _{1 \le i,j \le N} f_{ij}^2 \\ \le \;&I_{N-2} \, |\Lambda | \Big [ 2 \lambda _\ell \int \chi _\ell (x) f_\ell ^2 (x) \textrm{d}x \Big ] \\  &+ \sum _{m_1 = 0}^{M} (-1)^{m_1} {N-2 \atopwithdelims ()m_1} \sum _{m_2 = 0}^{M - m_1} (-1)^{m_2} {N-2-m_1 \atopwithdelims ()m_2} \chi (m_1 + m_2 \ge 1) \\  &\times \int V_{12} f_{12}^2 u_{1,3} \dots u_{1,m_1+2} u_{2,m_1+3} \dots u_{2, m_1 + m_2 + 2} \prod _{3 \le i < j \le N} f_{ij}^2 \\  &+ C \rho \mathfrak {a} (\rho \mathfrak {a}^2 \ell ) I_{N} / N \,. \end{aligned}\nonumber \\ \end{aligned}$$

Proceeding by induction we claim that, for every $h \in \mathbb {N}$, $h \ge 2$,

3.11 $$\begin{aligned} \begin{aligned} \int V_{12} \prod _{1 \le i<j \le N} f_{ij}^2 \le \;&|\Lambda | \Big [ 2\lambda _\ell \int \chi _\ell (x) f^2_\ell (x) \text {d}x \Big ] \Big [ I_{N-2} + \sum _{k=3}^h \alpha _k I_{N-k} \Vert u_\ell \Vert _1^{k-2} \Big ] \\  &+ \int V_{12} f_{12}^2 \, \beta _h \prod _{h+1 \le i < j \le N} f_{ij}^2\\  &+ C \rho \mathfrak {a}^2 \ell ^{-1} \sum _{j=2}^h (C \rho \mathfrak {a} \ell ^2)^{j-2} I_N / N \end{aligned} \end{aligned}$$

where we define that

$$\begin{aligned} \begin{aligned} \alpha _k&= \; \sum _{m_1=0}^M (-1)^{m_1} {N-2 \atopwithdelims ()m_1} \dots \hspace{-.2cm}\sum _{m_{k-1} = 0}^{M-m_1-\dots - m_{k-2}} (-1)^{m_{k-1}} {N-2-m_1-\dots -m_{k-2} \atopwithdelims ()m_{k-1}} \\&\quad \times \Big [ \prod _{j=2}^{k-2} \chi (m_1 + \dots + m_j \ge j-1) \Big ] \, \chi (m_1 + \dots + m_{k-1} = k-2) \end{aligned} \end{aligned}$$

and

$$ \begin{aligned} \beta _h&= \sum _{m_1=0}^M (-1)^{m_1} {N-2 \atopwithdelims ()m_1} \dots \sum _{m_h = 0}^{M-m_1-\dots - m_{h-1}} (-1)^{m_h} {N-2-m_1-\dots -m_{h-1} \atopwithdelims ()m_{h}} \\  &\quad \times \Big [ \prod _{j=2}^h \chi (m_1 + \dots + m_j \ge j-1) \Big ] \prod _{j_1 =3}^{m_1+2} u_{1,j_1} \prod _{j_2 =m_1+3}^{m_1+m_2+2} u_{2,j_2} \dots \prod _{j_h =m_1+\dots + m_{h-1} +3}^{m_1+ \dots +m_h +2} u_{h,j_h}\,. \end{aligned}$$

The coefficient $\alpha _k$ counts the number of contributions, arising in the expansion of the variables $x_1, \ldots , x_k$, which are disentangled from the remaining Bijl-Dingle-Jastrow factor (involving variables $x_{k+1}, \dots , x_N$) and contain no loops. On the other hand, the coefficient $\beta _h$ is associated with terms, produced in the expansion in the variables $x_1, \ldots , x_h$, that contain no loop but are still entangled with the remaining Bijl–Dingle–Jastrow factor (as follows from the condition $m_1 + \dots +m_h \ge h-1$). Notice that, by definition, $\beta _h$ is a sum of functions depending at least on the variables $x_1, . . . , x_{h+1}$. More precisely, the term with the indices $m_1,...,m_h$ depends on the variables $x_1,...,x_{m_1+\dots +m_{h+2}}$.

The bound (3.10) shows the validity of (3.11) with $h=2$, since $\rho \mathfrak {a}^2 \ell = \mathfrak {a} \ell ^{-1} (\rho \mathfrak {a} \ell ^2) \le \mathfrak {a} \ell ^{-1}$. To show the induction step we start from the bound (3.11) and, in the term proportional to $\beta _h$, we expand the dependence of the Bijl–Dingle–Jastrow factor on the $x_{h+1}$ variable, similarly as we did in (3.8). We obtain

3.12 $$\begin{aligned} \begin{aligned} \int V_{12}&f_{12}^2 \beta _h \prod _{h+1 \le i< j \le N} f_{ij}^2 \\ \le \;&\sum _{m_1=0}^M (-1)^{m_1} \left( {\begin{array}{c}N-2\\ m_1\end{array}}\right) \dots \sum _{m_h=0}^{M-m_1-\dots -m_{h-1}} (-1)^{m_h} \left( {\begin{array}{c}N-2-m_1-\dots -m_{h-1}\\ m_h\end{array}}\right) \\&\quad \times \left[ \prod _{j=2}^h \chi (m_1+\dots +m_j \ge j-1) \right] \sum _{m_{h+1} = 0}^{M-m_1-\dots - m_h} (-1)^{m_{h+1}} \sum _{h+2 \le r_1< \dots< r_{m_{h+1}} \le N} \\  &\quad \times \int V_{12} f_{12}^2 \prod _{j_1=3}^{m_1+2} u_{1,j_1} \dots \prod _{j_h = m_1+\dots +m_{h-1} + 3}^{m_1 + \dots + m_h +2} u_{h, j_h} \prod _{j=1}^{m_{h+1}} u_{h+1, r_j} \, \prod _{h+2 \le i < j \le N} f_{ij}^2 \,. \end{aligned}\nonumber \\ \end{aligned}$$

**Fig. 1:**
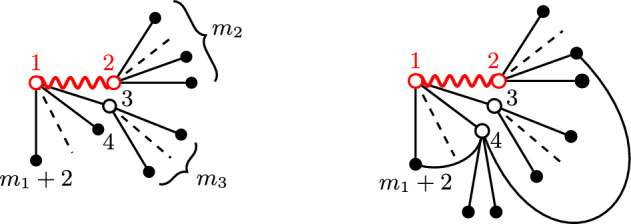
Graphical representation of two possible outputs of the expansion described between Eqs. (3.7) and (3.11). Nodes represent particles’ labels, the edge (*i*, *j*) represents a factor $u_{i,j}$ and the wiggly line stands for $V_{12}f^2_{12}$. On the l.h.s. a diagram representing one term in $\beta _3$, *i.e. *a term without loops obtained by expanding the $x_1, x_2$ and $x_3$-dependence in the Bijl–Dingle–Jastrow factor (expanded variables are denoted by empty nodes). On the r.h.s. one of the terms contributing to $\mathcal {E}_\mathrm{{loops}, 4}$: a diagram with $k=2$ loops which is obtained from the previous one by expanding the $x_4$-dependence

As we did above, we separate terms with no loops from terms with at least one loop (*i.e. *terms with an index $j\in \{ 1, \ldots , m_{h+1}\}$ such that $r_j \in \{h+2, \ldots , m_1+\ldots +m_{h} +2\}$). We decompose the contribution arising from terms without loops writing $1 = \chi (m_1 + \dots + m_{h+1} \ge h) + \chi (m_1 + \dots + m_{h+1} = h-1)$ (we can restrict our attention to the support of $\chi (m_1 + \dots + m_h \ge h-1)$). In particular the condition $\chi (m_1 + \dots + m_{h+1} = h-1)$ guarantees that the Bijl–Dingle–Jastrow factor is disconnected from the observable and all the $u_\ell$ factors. We conclude that

3.13 $$\begin{aligned} \begin{aligned} \int V_{12} f_{12}^2 \beta _h \prod _{h+1 \le i< j \le N} f_{ij}^2 \le \;&|\Lambda | \Big [ 2\lambda _\ell \int \chi _\ell (x) f^2_\ell (x) \textrm{d}x \Big ] \alpha _{h+1} I_{N-(h+1)} \Vert u \Vert _1^{h-1} \\  &+ \int V_{12} f_{12}^2 \, \beta _{h+1} \prod _{h+2 \le i < j \le N} f_{ij}^2 + \mathcal {E}_{\text {loops},h+1}, \end{aligned} \nonumber \\ \end{aligned}$$

where $\mathcal {E}_{\text {loops},h+1}$ denotes the contribution from terms on the r.h.s. of (3.12) with at least one loop (see Fig.1 for a graphical representation of some contributions to the iterative expansion described so far). To conclude the proof of Eq. (3.11) it remains to bound $\mathcal {E}_{\text {loops},h+1}$. Consider, for fixed $m_1, \ldots , m_{h+1}$, the term on the r.h.s. of (3.12) associated with the indices $(r_1, \ldots , r_{m_{h+1}})$, assuming that $r_{\alpha _1}, \dots r_{\alpha _k}$ close *k* loops, with $1 \le k \le \min (m_1+ \dots + m_h, m_{h+1})$, while the other $m_{h+1}-k$ variables are fresh. Choose one of the *k* loops, say the one linked with $r_{\alpha _1}$, denote by *s* its length (by construction, $s\ge 3$), and say it includes the edge (1, 2) (loops that do not involve the edge (1, 2) can be handled similarly). To bound the contribution of the integral associated with this choice of $(r_1, \dots , r_{m_{h+1}})$, we estimate $u_{h+1, r_{\alpha _j}}$ in $L^\infty$, for all $j=2,\dots , k$. After eliminating the dependence of the Bijl–Dingle–Jastrow function on their variables, we can then bound the remaining $m_1+\dots +m_{h+1} - (k-1) - (s -1)$ factors of $u_\ell$ that are not in the loop linked with $r_{\alpha _1}$ in $L^1$ (recall that we assumed the edge (1, 2) to be part of the loop; hence, the loop involves only $(s-1)$ factors of $u_\ell$). After appropriate renaming of the integration variables, this term can be estimated by

$$\begin{aligned} \begin{aligned} \Big | \int V_{12} f_{12}^2&\prod _{j_1=3}^{m_1+2} u_{1,j_1} \dots \prod _{j_h = m_1+\dots +m_{h-1} + 3}^{m_1 + \dots + m_h +2} u_{h, j_h} \prod _{j=1}^{m_{h+1}} u_{h+1, r_j} \, \prod _{h+2 \le i < j \le N} f_{ij}^2 \Big | \\ \le \;&C \Vert u_\ell \Vert _\infty ^{k-1} \Vert u_\ell \Vert _1^{m_1+ \dots +m_{h+1} +2 - k - s} I_{N- (m_1 + \dots +m_{h+1} + 2 - k)} \\  &\quad \times \int V_{12} f_{12}^2 u_{2,3} u_{3,4} \dots u_{s-1,s} u_{1,s} \text {d}x_1 \dots \text {d}x_s\,. \end{aligned} \end{aligned}$$

With Lemma 2.1, we can bound $\Vert u_\ell \Vert _\infty \le 1$, $\Vert u_\ell \Vert _1 \le C \mathfrak {a} \ell ^2$ and

$$ \begin{aligned} \int V_{12} f_{12}^2 u_{2,3}&u_{3,4} \dots u_{s-1,s} u_{1,s} \textrm{d}x_1 \dots \textrm{d}x_s \\  &\le C^s \frac{\mathfrak {a}^s |\Lambda |}{\ell ^3} \int \chi _\ell (y_1 + \dots + y_{s-1}) \prod _{j=1}^{s-1} \frac{\chi _\ell (y_j)}{|y_j|} \textrm{d}y_1 \dots \textrm{d}y_{s-1} \\  &\le C^s \mathfrak {a}^s \ell ^{2(s-1)-3} |\Lambda | \,. \end{aligned} $$

Taking into account that $s \le m_1 + \dots + m_{h+1} +2 - k$ and using (3.6), this leads to

$$\begin{aligned} \begin{aligned} \Big | \int V_{12} f_{12}^2 \prod _{j_1=3}^{m_1+2} u_{1,j_1} \dots&\prod _{j_h = m_1+\dots +m_{h-1} + 3}^{m_1 + \dots + m_h +2} u_{h, j_h} \prod _{j=1}^{m_{h+1}} u_{h+1, r_j} \, \prod _{h+2 \le i < j \le N} f_{ij}^2 \Big | \\ \le \;&C \mathfrak {a}^2 \ell ^{-1} (C \mathfrak {a} \ell ^2)^{m_1+ \dots +m_{h+1}- k} |\Lambda | I_{N- (m_1 + \dots +m_{h+1} + 2 - k)} \\ \le \;&C \rho \mathfrak {a}^2 \ell ^{-1} (C \mathfrak {a} \ell ^2/|\Lambda |)^{m_1+ \dots +m_{h+1}- k} I_N / N\,. \end{aligned} \end{aligned}$$

Thus, counting the number of terms on the r.h.s. of (3.12) producing *k* loops, we can estimate

$$ \begin{aligned}&N \mathcal {E}_{\text{ loops },h+1} \\  &\quad \le \; C \rho \mathfrak {a}^2 \ell ^{-1} \sum _{m_1=0}^M \left( {\begin{array}{c}N-2\\ m_1\end{array}}\right) \dots \\  &\qquad \sum _{m_h=0}^{M-m_1-\dots -m_{h-1}} \left( {\begin{array}{c}N-2 -\dots -m_{h-1}\\ m_h\end{array}}\right) \chi (m_1+\dots +m_h \ge h-1) \\  &\qquad \times \sum _{m_{h+1} = 0}^{M-m_1-\dots - m_h} \sum _{k=1}^{\min (m_{h+1}, m_1 + \dots + m_h)} \left( {\begin{array}{c}m_1 + \dots + m_h\\ k\end{array}}\right) \\  &\qquad \qquad \times \left( {\begin{array}{c}N-2 -m_1-\dots - m_h\\ m_{h+1} - k\end{array}}\right) (C \mathfrak {a} \ell ^2/|\Lambda |)^{m_1+ \dots +m_{h+1}- k} I_N \\  &\quad \le \; C \rho \mathfrak {a}^2 \ell ^{-1} \sum _{m_1=0}^M \;\dots \sum _{m_h=0}^{M-m_1-\dots -m_{h-1}} \chi (m_1+\dots +m_h \ge h-1)\\  &\qquad \sum _{k=1}^{m_1 + \dots + m_h} \left( {\begin{array}{c}m_1 + \dots + m_h\\ k\end{array}}\right) \\  &\qquad \times \sum _{m_{h+1} = k}^{M-m_1-\dots - m_h} \frac{N^{m_1+\dots +m_{h+1}-k}}{m_1! \dots m_h! (m_{h+1} -k)!} (C \mathfrak {a} \ell ^2/|\Lambda |)^{m_1+ \dots +m_{h+1}- k} I_N\,.\end{aligned} $$

Switching variables $m_{h+1} \rightarrow m_{h+1} - k$, we find

$$ \begin{aligned} N&\mathcal {E}_{\text {loops},h+1} \\ \le \;&C \rho \mathfrak {a}^2 \ell ^{-1} \sum _{m_1=0}^M \dots \sum _{m_h=0}^{M-m_1-\dots -m_{h-1}} \chi (m_1+\dots +m_h \ge h-1)\\  &\sum _{k=1}^{m_1+\dots + m_h} \left( {\begin{array}{c}m_1 + \dots + m_h\\ k\end{array}}\right) \\&\times \sum _{m_{h+1} = 0}^{M-m_1-\dots - m_h-k} \frac{1}{m_1! \dots m_h! m_{h+1}!} (C \rho \mathfrak {a} \ell ^2)^{m_1+ \dots +m_{h+1}} I_N \,. \end{aligned} $$

Next, we bound the sum over $m_{h+1}$ by $\exp (C\rho \mathfrak {a} \ell ^2) \le C$ and subsequently the sum over *k* by $2^{m_1 + \dots + m_h}$. Thus, we arrive at

$$ \begin{aligned} N \mathcal {E}_{\text{ loops },h+1}&\le \; C \rho \mathfrak {a}^2 \ell ^{-1} \sum _{m_1=0}^M \dots \sum _{m_h=0}^{M-m_1-\dots -m_{h-1}} \frac{ \chi (m_1+\dots +m_h \ge h-1) }{m_1! \dots m_h!}\\  &\qquad \times (C \rho \mathfrak {a} \ell ^2)^{m_1+ \dots +m_{h}} I_N \\  &\le \; C \rho \mathfrak {a}^2 \ell ^{-1} (C \rho \mathfrak {a} \ell ^2)^{h-1} I_N \,. \end{aligned} $$

Inserting in (3.13) and then plugging the resulting bound in (3.11), we complete the induction step, namely we prove (3.11), with *h* replaced by $h+1$. This proves the validity of (3.11), for all $h \in \mathbb {N}$, $h \ge 2$.

Choosing now $h=M$ in (3.11), we conclude that

3.14 $$\begin{aligned} \int V_{12} \prod _{1 \le i< j \le N} f_{ij}^2&\le \; |\Lambda | \Big [ 2\lambda _\ell \int \chi _\ell (x) f_\ell ^2 (x) \text {d}x \Big ] \Big [ I_{N-2} + \sum _{k=3}^M \alpha _k I_{N-k} \Vert u_\ell \Vert _1^{k-2} \Big ] \nonumber \\  &\quad + \int V_{12} f_{12}^2 \, \beta _M \prod _{M+1 \le i < j \le N} f_{ij}^2 \nonumber \\  &\qquad + C \rho \mathfrak {a}^2 \ell ^{-1}\sum _{j=2}^M (C \rho \mathfrak {a} \ell ^2)^{j-2} I_N / N \, . \end{aligned}$$

The integral containing $\beta _M$ cannot be computed explicitly (some of the variables are still entangled with the Bijl–Dingle–Jastrow factor). With the definition of $\beta _M$, and using the bound $\Vert u_\ell \Vert _1 \le C \mathfrak {a} \ell ^2$, following from Lemma 2.1, we can estimate its absolute value by

$$ \begin{aligned}&\Big | \int V_{12} f_{12}^2 \beta _M \prod _{M+1 \le i < j \le N} f_{ij}^2 \Big | \\  &\le C \; \lambda _\ell \ell ^3|\Lambda | \sum _{m_1=0}^M \dots \sum _{m_M=0}^{M-m_1- \dots - m_{M-1}} \chi (m_1 + \dots +m_M \ge M-1) \\  &\quad \times \frac{N^{m_1+\dots + m_M}}{m_1! \dots m_M!} \Vert u_\ell \Vert _1^{m_1 + \dots +m_M} I_{N-(m_1+ \dots + m_M+2)}\,. \end{aligned} $$

Taking into account the range of $m_1, \ldots , m_M$, we decompose $\chi (m_1 + \dots +m_M \ge M-1) = \chi (m_1 + \dots + m_M = M-1) + \chi (m_1 + \dots + m_M = M)$. We find

3.15 $$\begin{aligned} \begin{aligned} \Big | \int V_{12} f_{12}^2 \beta _M \prod _{M+1 \le i < j \le N} f_{ij}^2 \Big |&\le \; C \lambda _\ell \ell ^3|\Lambda | N^{M-1} \Vert u_\ell \Vert _1^{M-1} I_{N-(M+1)} e^M\\  &\quad + C \lambda _\ell \ell ^3|\Lambda | N^{M} \Vert u_\ell \Vert _1^{M} I_{N-(M+2)} e^M \\  &\le \; C \rho \mathfrak {a} \left[ (C \rho \mathfrak {a} \ell ^2 )^{M-1} +(C \rho \mathfrak {a} \ell ^2 )^M \right] I_N/ N \\  &\le C \rho \mathfrak {a} (C \rho \mathfrak {a} \ell ^2 )^{M-1} I_N / N\,. \end{aligned} \end{aligned}$$

We conclude that

3.16 $$\begin{aligned} \begin{aligned}&\int V_{12} \prod _{1 \le i < j \le N} f_{ij}^2 \le \; |\Lambda | \Big [ 2 \lambda _\ell \int \chi _\ell (x) f_\ell ^2 (x) \text {d}x \Big ] \Big [ I_{N-2} + \sum _{k=3}^M \alpha _k I_{N-k} \Vert u_\ell \Vert ^{k-2} \Big ] \\  &\quad +C \rho \mathfrak {a} (C \rho \mathfrak {a} \ell ^2)^{M-1} \, I_N / N\\  &\quad + C \rho \mathfrak {a}^2 \ell ^{-1} \sum _{j=2}^M (C \rho \mathfrak {a} \ell ^2)^{j-2} I_N / N\,. \end{aligned} \end{aligned}$$

Similarly, we can bound the denominator on the l.h.s. of (2.3) by

3.17 $$\begin{aligned} \begin{aligned} I_N&= \int \prod _{1 \le i < j \le N} f_{ij}^2 \ge \; |\Lambda | \Big [ \int f_\ell ^2 (x) \text {d} x \Big ] \Big [ I_{N-2} + \sum _{k=3}^{M} \alpha _k I_{N-k} \Vert u_\ell \Vert _1^{k-2} \Big ] \\  &\quad - C (C \rho \mathfrak {a} \ell ^2)^{M-1} \, I_N - C \mathfrak {a} \ell ^{-1} \sum _{j=2}^M (C \rho \mathfrak {a} \ell ^2)^{j-2} I_N \,. \\ \end{aligned}\nonumber \\ \end{aligned}$$

To prove this estimate, we proceed as in the derivation of (3.16), replacing *V* with 1. Since we need here a lower rather than an upper bound, in the first step we use (3.3) cutting the expansion at order $M+1$. As we did in the proof of (3.14), we then iterate $h=M$ times (despite the fact that we now cut expansions of the Bijl–Dingle–Jastrow function at order $M+1$, rather than *M*) using (3.2) and (3.3) depending on the parity of the order at which we stop the expansion. Proceeding as in (3.15) to bound terms in which the integral cannot be performed explicitly, we arrive at (3.17), with the coefficients $\alpha _k$ replaced by

$$\begin{aligned} \begin{aligned} \widetilde{\alpha }_k =&\sum _{m_1=0}^{M+1} (-1)^{m_1} {N-2 \atopwithdelims ()m_1} \,\dots \hspace{-.2cm}\sum _{m_{k-1} = 0}^{M+1-m_1-\dots - m_{k-2}} (-1)^{m_{k-1}} {N-2-m_1-\dots -m_{k-2} \atopwithdelims ()m_{k-1}} \\  &\hspace{1cm} \times \Big [ \prod _{j=2}^{k-2} \chi (m_1 + \dots + m_j \ge j-1) \Big ] \, \chi (m_1 + \dots + m_{k-1} = k-2)\end{aligned} \end{aligned}$$

for $k=1,\dots , M$. It is however easy to check that, due to the characteristic function $\chi (m_1 + \dots + m_{k-1} = k-2)$, the value of $\widetilde{\alpha }_k$ does not change if, on the r.h.s., we replace $M+1$ by *M*; in other words, $\widetilde{\alpha }_k = \alpha _k$, which leads to (3.17).

From (3.17), we obtain

$$\begin{aligned}  &   I_N \ge |\Lambda | \Big [ \int f_\ell ^2 (x) \text {d}x \Big ] \Big [ I_{N-2} + \sum _{k=3}^{M} \alpha _k I_{N-k} \Vert u_\ell \Vert _1^{k-2} \Big ]\\    &   \qquad \qquad \times \Big [ 1 - C (C\rho \mathfrak {a} \ell ^2)^{M-1} - C \mathfrak {a} \ell ^{-1} \sum _{j=2}^M (C \rho \mathfrak {a} \ell ^2)^{j-2} \Big ]\end{aligned}$$

where we used (3.6) to absorb the error terms on the second line of (3.17). Combining with (3.16), we arrive at (recall from (3.1) that $\ell = c (\rho \mathfrak {a})^{-1/2}$ so that $\mathfrak {a} / \ell \le C (\rho \mathfrak {a}^3)^{1/2} \ll 1$)

$$ \begin{aligned}&\frac{N}{2} \frac{\int V_{12} \prod _{i<j}^N f_{ij}^2}{I_N} \le \; N \frac{ \lambda _\ell \int \chi _\ell (x) f_\ell ^2 (x) \textrm{d}x}{\int f_\ell ^2 (x) \textrm{d}x} \\&\quad \left[ 1 + C (C \rho \mathfrak {a} \ell ^2)^{M-1} + C \mathfrak {a} \ell ^{-1} \sum _{j=2}^M (C \rho \mathfrak {a} \ell ^2)^{j-2} \right] \\  &\quad + C \rho \mathfrak {a} (C \rho \mathfrak {a} \ell ^2 )^{M-1} + C \rho \mathfrak {a}^2 \ell ^{-1} \sum _{j=2}^M (C \rho \mathfrak {a} \ell ^2)^{j-2} \,. \end{aligned} $$

The simplification of the factor $\big [I_{N-2} + \sum _{k=3}^{M} \alpha _k I_{N-k} \Vert u \Vert _1^{k-2} \big ]$ corresponds to the cancellation of tree diagrams that we mentioned at the end of Sect. 2. Notice that with our construction we only need to cancel trees with at most *M* vertices.

Using Lemma 2.1, we find

$$ \lambda _\ell \int \chi _\ell (x) f_\ell ^2 (x) \textrm{d}x \le 4\pi \mathfrak {a} \Big [ 1 + C \frac{\mathfrak {a}}{\ell } \Big ]\,. $$

Since moreover $\int f_\ell ^2 (x) \textrm{d}x \ge |\Lambda | - C \mathfrak {a} \ell ^2$, we conclude that

3.18 $$\begin{aligned} \begin{aligned}&\frac{N}{2} \frac{\int V_{12} \prod _{i<j}^N f_{ij}^2}{\int \prod _{i<j}^N f_{ij}^2} \le 4\pi \rho \mathfrak {a}\\&\quad \left[ 1 + C (C \rho \mathfrak {a} \ell ^2)^{M-1} + C \mathfrak {a} \ell ^{-1} \sum _{j=2}^M (C \rho \mathfrak {a} \ell ^2)^{j-2} \right] \,. \end{aligned} \end{aligned}$$

Choosing $\ell = c (\rho \mathfrak {a})^{-1/2}$ as indicated in (3.1), with $c > 0$ so small that, on the r.h.s. of the last equation, $C \rho \mathfrak {a} \ell ^2 \le 1/2$, and choosing then the even number $M \ge 1 + \log _2 (\rho \mathfrak {a}^3)^{-1/2}$, we obtain

$$\begin{aligned} \frac{N}{2} \frac{\int V_{12} \prod _{i<j}^N f_{ij}^2}{\int \prod _{i<j}^N f_{ij}^2} \le 4\pi \rho \mathfrak {a} \big [ 1 + C (\rho \mathfrak {a}^3)^{1/2} \big ] \, . \end{aligned}$$

## Proof of Proposition 2.3

We proceed here similarly as in the proof of Proposition 2.2. For this reason, we will skip some of the details. As in (3.1), we fix $\ell = c (\rho \mathfrak {a})^{-1/2}$ for a sufficiently small constant $c >0$.

Recalling the definition $u_{ij} = 1 - f_{ij}^2$ and the notation (3.5), we set

$$ \begin{aligned} \mathcal {E}&= \frac{N^2}{I_N} \int \nabla f_{13}^2 \cdot \nabla f_{23}^2 \, f_{12}^2 \prod _{j \ge 4} f_{1j}^2 f_{2j}^2 f_{3j}^2 \prod _{4 \le i< j \le N} f_{ij}^2 \\  &=\frac{N^2}{I_N} \int \nabla u_{1,3} \cdot \nabla u_{2,3} \, f_{12}^2 \prod _{j \ge 4} f_{1j}^2 f_{2j}^2 f_{3j}^2 \prod _{4 \le i < j \le N} f_{ij}^2\,. \end{aligned} $$

With Lemma 2.1 and with (3.6) we find

$$ N^2 \frac{I_{N-3}}{I_N} \int |\nabla u_{1,3}| |\nabla u_{2,3}| \, u_{1,2} \, \textrm{d}x_1 \textrm{d}x_2 \textrm{d}x_3 \le C \rho ^2 \mathfrak {a}^3 \ell , $$

and therefore (noticing that $\int \nabla u(x) \,\textrm{d}x=0$),

4.1 $$\begin{aligned} \Big |\mathcal {E}-\frac{N^2}{I_N} \int \nabla u_{1,3} \cdot \nabla u_{2,3} \prod _{r=4}^N f^2_{1r}f^2_{2r} f^2_{3r} \prod _{4\le i <j\le N} f^2_{ij} \Big | \le C \rho \mathfrak {a} (\rho \mathfrak {a}^2 \ell ) \,. \end{aligned}$$

Next, we expand the Bijl–Dingle–Jastrow factors, one variable after the other. Since here, in contrast with the proof of Proposition 2.2, the observable does not have a sign, when we stop an expansion we always have to estimate the error. We will use multiple times the inequality (3.4). Applying this bound to (4.1), we find

$$\begin{aligned} \begin{aligned} \Big |\mathcal {E}-&\frac{N^2}{I_N} \sum _{m_1=1}^M (-1)^{m_1} \left( {\begin{array}{c}N-3\\ m_1\end{array}}\right) \int \nabla u_{1,3} \cdot \nabla u_{2,3} \, u_{1,4}\dots u_{1,m_1+3}\prod _{r=4}^N f^2_{2r}f^2_{3r} \prod _{4\le i<j\le N}f^2_{ij} \Big |\\ \le \;&C \frac{C^M N^{M+3}}{(M+1)! I_N} \int |\nabla u_{1,3}|\,|\nabla u_{2,3}| \, u_{1,4}\dots u_{1, M+4} \prod _{4 \le i<j\le N} f^2_{ij} + C\rho \mathfrak {a} (\rho \mathfrak {a}^2 \ell ) \\ \le \;&C \frac{C^M N^{M+3}}{(M+1)! I_N} \Vert \nabla u_\ell \Vert _1^2 \,\Vert u_\ell \Vert _1^{M+1} |\Lambda | I_{N-(M+4)} + C \rho \mathfrak {a} (\rho \mathfrak {a}^2 \ell ) \\ \le \;&C \rho \mathfrak {a} (C \rho \mathfrak {a} \ell ^2)^{M+2} + C \rho \mathfrak {a} (\rho \mathfrak {a}^2 \ell ) , \end{aligned} \end{aligned}$$

where in the last step we estimated $\Vert \nabla u_\ell \Vert _1 \le C \mathfrak {a} \ell$, $\Vert u_\ell \Vert _1 \le C \mathfrak {a} \ell ^2$ and $I_N \ge 2^{-(M+4)} I_{N-(M+4)} |\Lambda |^{M+4}$ as follows from (3.6). Notice that the sum on the l.h.s. starts from $m_1 = 1$, because the contribution with $m_1 = 0$ vanishes (since $\int \nabla u_\ell (x) \text {d}x = 0$).

Let us now expand the $x_2$-dependence. We find

4.2 $$\begin{aligned} \Big | \mathcal {E}-&\frac{N^2}{I_N} \sum _{m_1=1}^M (-1)^{m_1} \left( {\begin{array}{c}N-3\\ m_1\end{array}}\right) \sum _{m_2 = 1}^{M-m_1} (-1)^{m_2} \sum _{4\le j_1<\dots< j_{m_2} \le N}\nonumber \\  &\quad \times \int \nabla u_{1,3} \cdot \nabla u_{2,3} \, u_{1,4} \dots u_{1, m_1+3} u_{2,j_1} \dots u_{2,j_{m_2}} \prod _{r=4}^N f^2_{3r} \prod _{4\le i<j\le N}f^2_{ij} \Big | \nonumber \\ \le \;&C \frac{N^2}{I_N} \sum _{m_1=1}^M \frac{N^{m_1}}{m_1!} \sum _{4\le j_{1}<\dots< j_{M+1-m_{1} }\le N} \nonumber \\  &\quad \times \int |\nabla u_{1,3}|\,|\nabla u_{2,3}| \, u_{1,4} \dots u_{1,m_1+3} u_{2,j_1}\dots u_{2,j_{M+1 -m_1}} \prod _{4\le i<j\le N}f^2_{ij} \nonumber \\&+ C \rho \mathfrak {a} (C\rho \mathfrak {a} \ell ^2)^{M+2} + C \rho \mathfrak {a} (\rho \mathfrak {a}^2 \ell )\,. \end{aligned}$$

Denoting by $0 \le k \le \min (m_1, M+1-m_1)$ the number of loops that are formed by the indices $j_1, \ldots , j_{M+1-m_1}$, we can bound the first term on the r.h.s. of (4.2) by

$$ \begin{aligned} C\,&\frac{N^2}{I_N} \sum _{m_1=1}^M \frac{N^{m_1}}{m_1!} \sum _{k=0}^{\min (m_1, M+1-m_1)} \left( {\begin{array}{c}m_1\\ k\end{array}}\right) \left( {\begin{array}{c}N-3-m_1\\ M+1-m_1 - k\end{array}}\right) \;I_{N-(M-k+4)} \\&\times \int |\nabla u_{1,3}| |\nabla u_{2,3}| \Big [ \prod _{j=4}^{k+3} u_{1,j} u_{2,j} \Big ] \, u_{1,k+4} \dots u_{1, m_1+3} u_{2, m_1+4} \dots u_{2, M-k+4} \, \textrm{d}x_1 \dots \textrm{d}x_{M-k+4} \\ \le \;&C \sum _{k=0}^{(M+1)/2} \frac{1}{k!} \sum _{m_1 = k}^{M+1-k} \frac{1}{(m_1-k)! (M+1-m_1- k)!} \,C^{M+3}\,\rho ^{M+3-k} (\mathfrak {a} \ell ^2)^{M+1-2k} \mathfrak {a}^{2k+2} \\&\hspace{2cm} \times \int \frac{\chi _\ell (x_1)}{|x_1|^2} \frac{\chi _\ell (x_2)}{|x_2|^2} \prod _{j=1}^k \frac{\chi _\ell (y_j)}{|y_j|} \frac{\chi _\ell (y_j + x_1 + x_2)}{|y_j + x_1 + x_2|} \textrm{d}x_1 \textrm{d}x_2 dy_1 \dots dy_k \\ \le \;&C \rho \mathfrak {a} \sum _{k=0}^{(M+1)/2} \frac{1}{k!} \sum _{m_1 = 0}^{M+1-2k} \frac{1}{m_1! (M+1-2k-m_1)!} (C \rho \mathfrak {a} \ell ^2)^{M+2-2k} (C \rho \mathfrak {a}^2 \ell )^k \\ \le \;&C \rho \mathfrak {a} \sum _{k=0}^{(M+1)/2} \frac{1}{k!} \frac{1}{(M+1-2k)!} (C \rho \mathfrak {a} \ell ^2)^{M+2-2k} (C \rho \mathfrak {a}^2 \ell )^k \\&\quad \le \frac{C}{(M+1)!}\rho \mathfrak {a} (C \rho \mathfrak {a} \ell ^2)^{M+2} + C \rho \mathfrak {a} (\rho \mathfrak {a}^2 \ell ) \end{aligned} $$

where we used Lemma 2.1, the bound $\sup _x \int \chi _\ell (y)/ (|y| |x+y| )\le C \ell$ and, in the last step, we distinguished the cases $k=0$ and $k > 0$ (and we exploited the smallness of $\rho \mathfrak {a} \ell ^2$, resulting from (3.1)). Next, we bound the contribution of terms with loops on the l.h.s. of (4.2) (*i.e. *contributions to the second term on the l.h.s. of (4.2) with at least one $r \in \{ 1, \ldots , m_2\}$ satisfying $j_r \in \{ 4, \ldots , m_1+3 \}$). Denoting by $k>0$ the number of loops we have, proceeding similarly as to the above,

$$ \begin{aligned}&\Big |\frac{N^2}{I_N} \sum _{m_1=1}^M (-1)^{m_1} \left( {\begin{array}{c}N-3\\ m_1\end{array}}\right) \sum _{m_2 = 1}^{M-m_1} (-1)^{m_2} \sum _{k=1}^{\min \{m_1,m_2\}}\left( {\begin{array}{c}m_1\\ k\end{array}}\right) \left( {\begin{array}{c}N-3-m_1\\ m_2-k\end{array}}\right) \\&\quad \times \int \nabla u_{1,3} \cdot \nabla u_{2,3} \,u_{1,4}\dots u_{1,k+3}u_{2,4}\dots u_{2,k+3}\\&\quad \times u_{1,k+4}\dots u_{1,m_1+3}u_{2,m_1+4}\dots u_{2,m_1+m_2+3-k}\prod _{r=4}^N f^2_{3r} \prod _{4\le i<j\le N}f^2_{ij} \Big |\\&\le C\rho {{\mathfrak {a}}}\sum _{m_1=1}^M\sum _{m_2=1}^{M-m_1}\sum _{k=1}^{\min \{m_1,m_2\}}\frac{1}{k!}\frac{1}{(m_2-k)!}(C\rho {{\mathfrak {a}}}\ell ^2)^{m_1+m_2+1-2k}(C\rho {{\mathfrak {a}}}^2\ell )^k\le C\rho {{\mathfrak {a}}}(\rho {{\mathfrak {a}}}^2\ell )\,. \end{aligned} $$

We arrive at

$$\begin{aligned}&\left| \mathcal {E} - \frac{N^2}{I_N} \sum _{m_1=1}^M (-1)^{m_1} \left( {\begin{array}{c}N-3\\ m_1\end{array}}\right) \sum _{m_2 = 1}^{M-m_1} (-1)^{m_2} \left( {\begin{array}{c}N-3-m_1\\ m_2\end{array}}\right) \right. \\  &\left. \qquad \times \int \nabla u_{1,3} \cdot \nabla u_{2,3} \, u_{1,4} \dots u_{1, m_1+3} u_{2,m_1+4} \right. \\  &\left. \qquad \qquad \dots u_{2, m_1+m_2+3} \prod _{r=4}^N f^2_{3r} \prod _{4\le i<j\le N}f^2_{ij} \right| \\  &\quad \le \;\frac{C}{(M+1)!} \rho \mathfrak {a} (C \rho \mathfrak {a} \ell ^2)^{M+2} + C \rho \mathfrak {a} (\rho \mathfrak {a}^2 \ell )\,.\end{aligned}$$

Proceeding inductively (similarly as in the proof of Proposition 2.2, with the simplification that, here, all terms that contain no loop and are not entangled with the Bijl–Dingle–Jastrow factor vanish), we find, after *M* iterations,

$$\begin{aligned}&\left| \mathcal {E}- \frac{N^2}{I_N} \sum _{m_1=1}^M \sum _{m_2=1}^{M-m_1} \dots \sum _{m_M = 0}^{M-m_1 - m_2-\dots -m_{M-1}} (-1)^{m_1+\dots +m_M}\right. \\  &\left. \qquad \times \left( {\begin{array}{c}N-3\\ m_1\end{array}}\right) \dots \left( {\begin{array}{c}N-3-m_1-\dots - m_{M-1}\\ m_M\end{array}}\right) \,\right. \\  &\left. \qquad \times \Big [ \prod _{j=5}^M \chi (m_1+ \dots + m_j \ge j-2) \Big ] \right. \\  &\left. \qquad \times \int \nabla u_{1,3} \cdot \nabla u_{2,3} \, \prod _{j_1=4}^{m_1+3} u_{1,j_1} \dots \prod _{j_M =m_1+\dots +m_{M-1} +4}^{m_1+\dots + m_M +3} u_{M,j_M} \prod _{M+1 \le i<j\le N}f^2_{ij} \right| \\  &\quad \le \;C \rho \mathfrak {a} (C \rho \mathfrak {a} \ell ^2)^{M+2} + C \rho \mathfrak {a} (\rho \mathfrak {a}^2 \ell ) \sum _{j=2}^M (C \rho \mathfrak {a} \ell ^2)^{j-2} \,. \end{aligned}$$

The cutoffs $\chi (m_1 + \dots + m_j \ge j-2)$ make sure that, in all summands, the observable is still entangled with the Bijl–Dingle–Jastrow function. After removing contributions with loops, the cutoffs can be inserted for free, because $\int \nabla u_\ell (x) \text {d}x = 0$.

Finally, estimating the absolute value of the sum on the l.h.s. of last equation by

$$ \begin{aligned} C \frac{N^2}{I_N} |\Lambda |&\sum _{m_1, \ldots , m_M = 0}^M \frac{N^{m_1 + \dots + m_M}}{m_1! \dots m_M!} \chi (M-2 \le m_1 +\dots + m_M \le M) \\  &\quad \times \Vert \nabla u_\ell \Vert _1^2 \Vert u_\ell \Vert _1^{m_1+ \dots + m_M} I_{N-(m_1+\dots + m_M +3)} \\ \le \;&C \rho \mathfrak {a} \, (C \rho \mathfrak {a} \ell ^2)^{M-1} \end{aligned} $$

we conclude that

$$\begin{aligned} |\mathcal {E}| \le C \rho \mathfrak {a} (C \rho \mathfrak {a} \ell ^2)^{M-1} + C \rho \mathfrak {a} (\rho \mathfrak {a}^2 \ell )\,. \end{aligned}$$

Recalling our choice of $\ell = c (\rho \mathfrak {a})^{-1/2}$, fixing $c > 0$ so small that $C \rho \mathfrak {a} \ell ^2 \le 1/2$ and subsequently choosing the integer $M > 1 + \log _2 (\rho \mathfrak {a}^3)^{-1/2}$, we obtain that

$$\begin{aligned} |\mathcal {E}| \le C \rho \mathfrak {a} (\rho \mathfrak {a}^3)^{1/2} \end{aligned}$$

for a sufficiently large constant $C >0$. This concludes the proof of Proposition 2.3.

## Data Availability

Data sharing not applicable to this article as no datasets were generated or analysed during the current study.
